# Nature Studies

**Published:** 1909-11-20

**Authors:** 


### The Practitioner's Relaxations.

### NATURE STUDIES.

By MAJOR, R.A.M.C.

On the Downs: a Successful Farmer—Bird Life at Close Quarters—A Paradise for Birds—The Peewit and Plover—Flies in the Frost.

My house is midway between two little villages, about one mile from each of them, and at the crossing of two great highways that run straight across the Downs, the one north and south, the other east and west. From my small domain no sign of either village can be seen, nor of human habitation, but that of my only neighbour. He lives in a trim red house just across the road. In the most rural of rural districts I rent a house and stabling and a three-acre paddock. My holding is an island in the ocean of territory of a man of great estate. This wealthy gentleman manages his estate generously, and himself farms some five thousand acres. The land is farmed on scientific lines with lavish outlay of capital; the cultivation is perfection; the stock the best obtainable; the cottages models; the game is strictly preserved, and the rabbits kept down to a low level. This estate is a standing example of the good use a good landlord with plenty of capital can make of land; and it pays, I understand, 4 per cent. on the capital expended. If so, it must bring in a large income.

Isolation from mankind gives me company of bird and beast. I do not see hawks, nor stoats, nor other vermin, for the preservation is too strict for them to thrive. Though I regret their absence, for I love to see the kestrel hover and to see the keen, wedge-shaped head and white throat of the stoat pop up from a rabbit burrow or through a meuse in a fence, still the preservation gives me other objects of interest. Only this fine October morning I sprang a cock pheasant from the rough grass of my paddock, and my servants reported last night that a pheasant, perhaps the same, was feeding beneath the kitchen window. Often as I dress I see two cock pheasants fighting at the edge of a cover to the east of the south running road, and hear the partridges calling in the clover field not fifty yards from my window. My domain and its borders teem with wild life. Because of the absence of furred and feathered foes and of the presence of many detached covers and plantations with cultivated fields between them, small birds abound.

And because of the vast spaces of the downland that favour their nesting, peewits are more plentiful than I have seen them elsewhere. Now that autumn is on us they are in large flocks in many of the fields within a mile of my house, and flocks fly daily across my paddock. I observe that flocks are feeding on the fallows, flocks are feeding on the downland, and others amongst the root crops and in the meadows. Now to me it seems odd that at the same times of the same days these birds should be seeking their food on such varying soils. Practically all partridges are in the stubbles at early morning and in the evening time, because it is there they seek their food. Equally certainly in the middle of warm days they will be found in the roots because the broad leaves give shelter from the sun and in dry bright days they will sun themselves, sheltered by the hedgerows, and dust themselves like hens on an ashheap. Given the same conditions of time and weather and most of the partridges will be found in the same sort of ground. But these flocks of peewits are at the same time and under the same conditions of weather on very varying ground. In all the places they frequent they must find suitable food, or they would not be there. Yet it can hardly be identical supply.

Since they are not persecuted by promiscuous gunners and seldom hear a shot fired, for the shooting is done at intervals and then confined to the more strictly game birds, the peewits here are very tame. I can ride almost into a flock any day. They will not rise till I am so near that I can distinguish between the plumage of the old birds and the birds of this year. It is pretty to watch them run along the ground and peck down on to their food, the head going down and the tail going up, for they move their bodies all in one piece as if hinged on to their leg bones. And even more beautiful to see are the evolutions of the flocks in the air. A flock moves as if by word of command, and keeps rank almost like a regiment. I think they must have discipline, for if not how could they wheel and rise and sink so certainly together. Then see how they throw forward scouts to spy out the land. The scout separates from the flock and goes over and scans the feeding ground. He wheels out, flaps away slowly, and gradually descends drifting towards the ground. Should he see a lurking gunner or other suspicious object he checks and wheels back and flaps hurriedly away to the flock, with haste, rising rapidly above shot reach. And all the flock, warned and alert to the danger, wheel and rise and fly steadily off to safer ground.

I began to write of my domain and digressed. Try back. The house faces south, so gets all the warmth of the sun from its rising to its setting, for no near trees shade the southern side. There is a verandah the length of this face. Now that the early mornings are frosty this verandah has great attraction for flies. So soon as the sun has power to warm the wall many come and sit on it and thaw their moribund tissues. I hate flies, but can pity them. It must be a dreadful life to freeze all night and thaw all day till exhaustion kills, or a wren or a sparrow gives more speedy end. It is most interesting to watch small birds hunting flies. Especially under thatched eaves, the sparrows hover against the wall and peck off the flies. Sometimes they cling to the wall and spread their tails against it to keep their balance; just as a woodpecker, with more efficient apparatus, does on the trunk of a tree. The wrens prefer to hunt low on the sunny side of smooth barked trees. It is queer that the wren, a bird *sui generis* in England, and by his looks so certainly meant for the tropics, should brave the English winter and thrive through the hardest frosts.

